# Supplementary material for: Drivers, Risk Factors and Dynamics of African Swine Fever Outbreaks, Southern Highlands, Tanzania
Source: Pathogens. 2020 Feb 25;9(3):155. doi: 10.3390/pathogens9030155 (PMC7157628; doi:10.3390/pathogens9030155)
Supplement: Supplementary file 1 [file pathogens-09-00155-s001.pdf]

pathogens

MDPI

Supplementary material

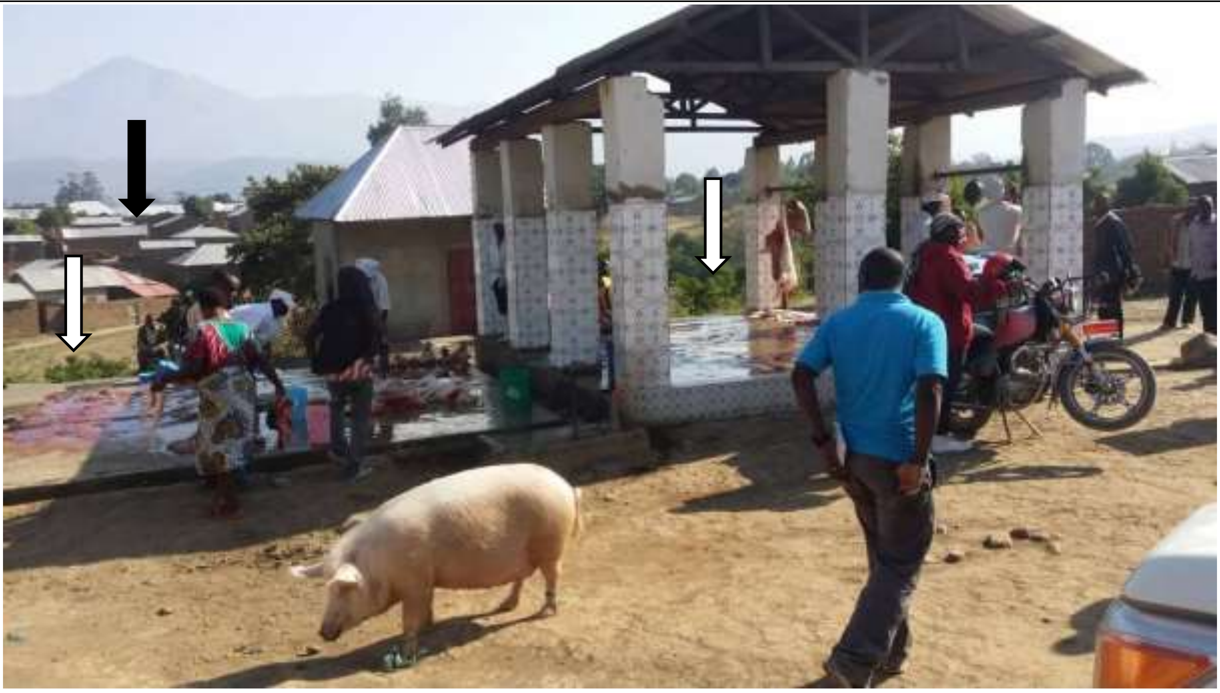

Plate 1a. Typical pig slaughter slab in the Southern Highlands, United Republic of Tanzania. Note that the slab is located upstream with many community downstream and a seasonal watercourse (stream) that serves the communities. Wastewater from the slaughter activities often overflow from the holding tanks and flow into and contaminate the stream. This may serve as source of pathogen for animals who drink routinely from the water.

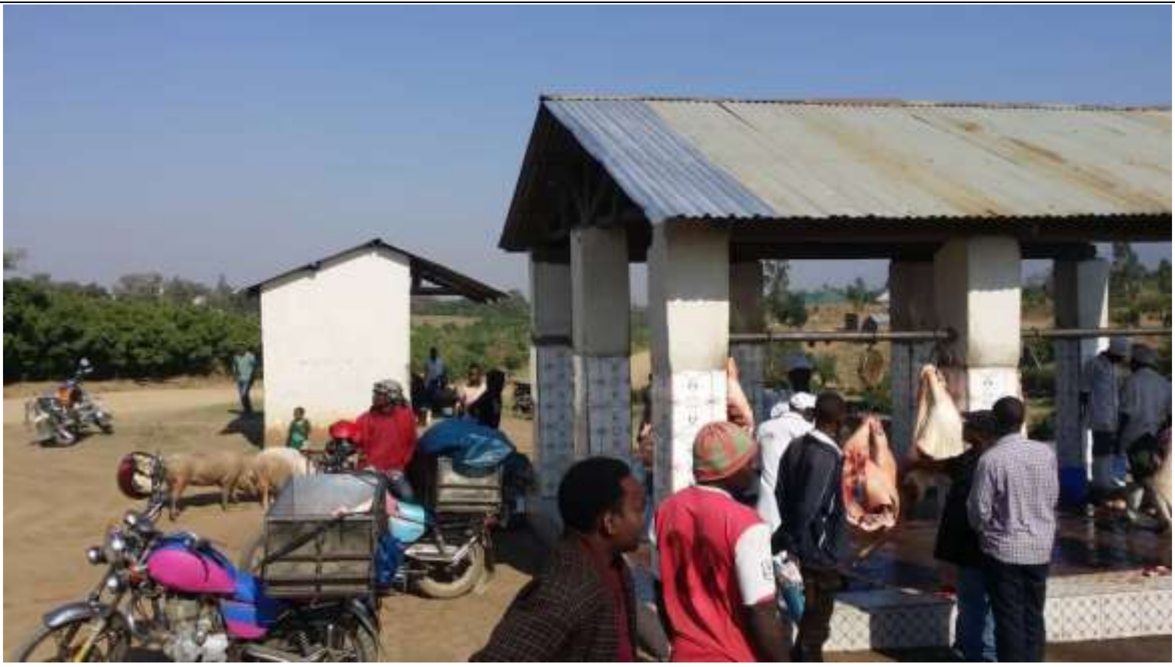

Plate 1b. The slaughter slab showing many ongoing activities including interactions between farmers, butchers and slaughter assistants, the transporter waiting with their motorcycles to transport the pork and pig products, a pig brought for slaughter interacting with a scavenging pig, and the veterinary inspector conducting a post-mortem inspection on the carcass before release.

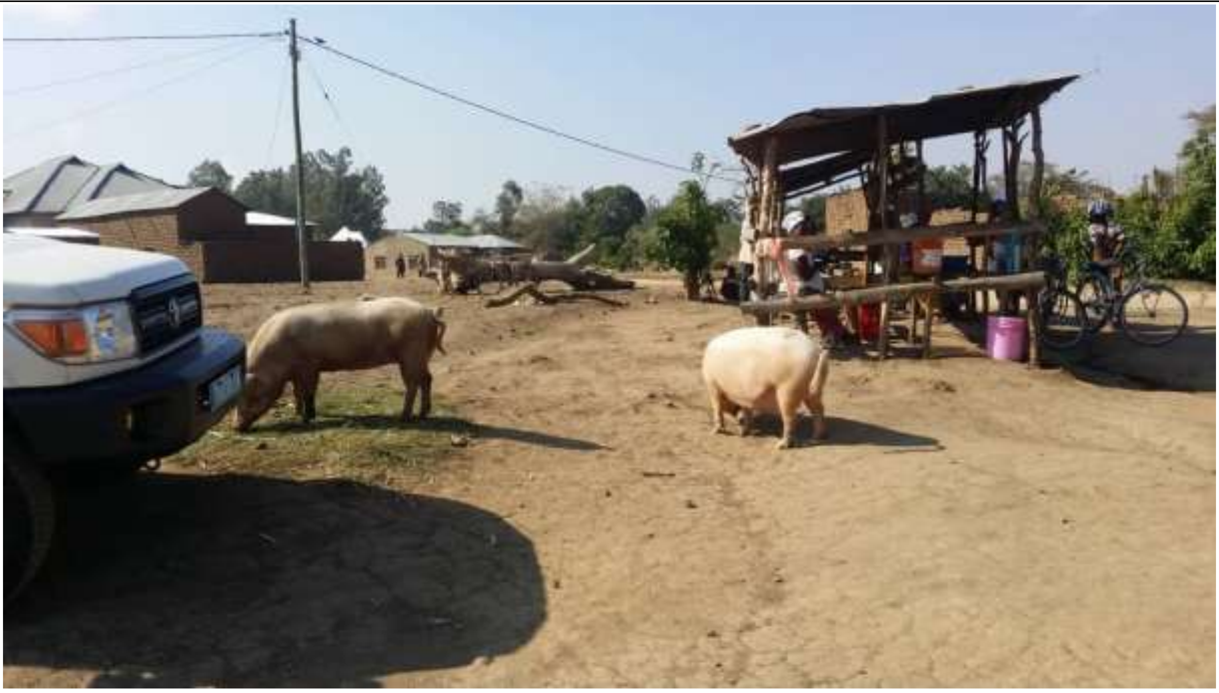

Plate 1c. Pigs, either scavenging or brought for slaughter were observed in the vicinity of the slaughter facility roaming freely and having contracts with remnants from the pig slaughter slabs. Where these pigs were not bought up for slaughter, they are kept for some days in the neighbourhood of the slaughter slabs and are later returned to farms of origin, potentially carrying infection back to the farms.

Supplementary Notes

Spatio-temporal distributions of outbreaks of ASF in the Southern Highlands, Tanzania

In November 2010, outbreaks of ASF were first reported in Kyela district of Mbeya region. Kyela district shares international boundaries with Malawi and legal and illegal trades of livestock occur across the borders. This outbreaks spread inwards and covered 7 districts of Mbeya region (Rungwe, Ileje, Mbarali, Mbeya Rural, Chunya, Mbozi and the City of Mbeya). Approximately 10 000 pigs died in the affected districts of Mbeya region and the outbreak was later brought under control. By February 2011 the disease was reported in the neighboring Ludewa and Makete districts, Iringa region, and by July 2011 the disease had spread to Njombe district of Iringa region. An estimated 1 300 pigs died in the Iringa region by mid-November 2011 when the outbreak was controlled. Concurrently with the outbreaks in Iringa, the Temeke, Ilala, and Kinondoni municipalities of Dar es Salaam region got infected with ASF around March 2011 with a loss of over 600 pigs by mid-November 2011. As from September 2011, further outbreaks were reported in Sumbawanga district, Rukwa region, again in the Southern Highlands, with the infection of six (6) villages in two divisions that border Mbozi district. In the Sumbawanga outbreaks, over 1 700 pigs died by November 2011 [10]. In 2017, another wave of outbreaks of ASF was reported in Sumbawanga (Figures 1 & 2).

In Chunya district, the index outbreak occurred in 2011 from spread of infections from outbreaks from villages of Songwe district (formerly Chunya district). The outbreaks followed the route of Songwe River which flows through Mbeya city into the Lake Rukwa. Subsequently, most villages along the river (including but not limited to Ifwenkenya, Kanga, Galula, Mbuyuni wards and wards in and around Chunya city) were all infected with massive losses of pigs. Subsequent outbreaks were recorded in other wards of Chunya in 2012 and 2014, with a less severe economic impacts, less geographical coverage, lesser mortality and reduced spread. A third cycle of ASF outbreaks have occurred in 2017 with similar impacts to those of 2014 outbreaks. In 2019, outbreaks have resurged in Sangambi and Chalangwa wards of Chunya district (Figures 1 & 2).

ASF occurred in Mbeya district, first in early 2017 but the origin was difficult to trace immediately. The disease started and became widespread during the rains of 2017 (November/December 2017) following flooding in different parts of the district. An unaccounted but large numbers of pigs died during this outbreak and no compensation was paid. Specifically, outbreaks were recorded in the following wards: Nsalala, Utengule Usongwe, Bonde la Songwe, Iwindi, Igale, Iyunga Mapinduzi, Santilya, Ilembo, Isuto, Ijombe, Tembela, Swaya and Mshewe. It should be noted that Mbeya district has the major livestock market (Pig Market) in Chang'ombe village, Mjele ward. Because this market is used by pig farmers from other districts, including but not limited to Chunya and Songwe, outbreaks in Mbeya was likely disseminated to these other districts inadvertently by long distant transportations and pig trades (Figures 1 & 2).

Similarly, in the Rukwa region, outbreaks have been sustained since 2011 and appeared again in 2014 and 2017. Specifically, the index ASF outbreak in domestic pigs was reported in Sumbawanga district in 2011. This was confirmed to have originated from infected pigs from villages of the bordering Momba district; subsequent outbreaks prevailed and spread especially on the Kaoze and Miangulua wards with the death of 2 378 pigs. A second wave of outbreaks occurred in 2013/2014, primarily at the Nkasi district involving Namanyere, Nkomolo, Kirando, Kipiri, Korongwe and Kabwe wards in which over 1 100 pigs died. In 2017, the last reported wave was reported in a multiple wards and villages' infections in Kalambo

and Sumbawanga districts where over 3 400 pigs reportedly died. Both home slaughter and the use of slaughter slabs prevailed in these communities particularly in Rukwa and Katavi. It is not yet known whether the ongoing outbreaks of 2019 involves the Rukwa region (Figures 1 & 2).

Ileje district experienced ASF, first in November 2010 with the death of 571 pigs in Isongole, Chitete, Itumba and Mbebe wards. It was suspected that the virus was introduced from Mbeya Rural which was also infected from Kyela, a boundary district with Malawi. The Kyela outbreaks were associated with outbreaks in Karonga and Chitipa districts of Malawi which was infected at the same time and shared trade, market access and similar management system with districts in the Southern Highlands zone of Tanzania. By August 2011, another wave of outbreaks occurred in Chitete ward at the borders of Malawi, with only 43 pigs reported dead, although anecdotal evidence suggested that many more pigs died and went unreported. A repeat outbreaks occurred in March 2016 in Ileje district (Msia, Chitete and Isongole wards), just three weeks after the disease was reported in the neighboring district of Chitipa, Malawi. This later spread to Itumba ward within three weeks. It will appear that the disease has now assumed an asymptomatic or less pathogenic forms as the death patterns were reduced compared with what was observed in previous outbreaks. In 2017, specifically from March to June, ASF infected Ileje district again with deaths of pigs (at least 67 pigs) mainly in the Isongole ward (first reported infection) and Itumba ward (Figures 1 & 2).

The current outbreaks (2019), particularly in some Chunya communities appear to have originated from a town in the neighboring country, Karonga area of Malawi, and is thought to have spread across the border into Kyela, Tanzania from where further dissemination into the inlands were recorded. It is suspected that Songwe and Tunduma, two districts that share borders with Zambia, and Kyela, a district that share border with Malawi are high surveillance zones for ASF, and may have ongoing infections.
